# Total Tumor Load of mRNA Cytokeratin 19 in the Sentinel Lymph Node as a Predictive Value of Axillary Lymphadenectomy in Patients with Neoadjuvant Breast Cancer

**DOI:** 10.3390/genes12010077

**Published:** 2021-01-08

**Authors:** Karla B. Peña, Amillano Kepa, Alba Cochs, Francesc Riu, David Parada, Josep Gumà

**Affiliations:** 1 Department of Pathology, Hospital Universitari de Sant Joan, Institut d’Investigació Sanitària Pere Virgili, Universitat Rovira i Virgili, 43204 Reus, Spain; karlabeatriz.pena@grupsagessa.com (K.B.P.); friu@grupsagessa.com (F.R.); 2 Department of Oncology, Hospital Universitari de Sant Joan, Institut d’Investigació Sanitària Pere Virgili, Universitat Rovira i Virgili, 43204 Reus, Spain; kamillano@grupsagessa.com (A.K.); alba.cochs@grupsagessa.com (A.C.)

**Keywords:** breast cancer, sentinel lymph node, nucleic acid amplification, OSNA, total tumor load, neoadjuvant chemotherapy, axillary lymphadenectomy

## Abstract

Although sentinel lymph node biopsy (SLNB) has proved to be able to diagnose axillary lymph node status safely and reliably, there is still not enough evidence to suggest that it can be used in patients who have undergone neoadjuvant chemotherapy (NAC) for lymph node-sparing surgery. The present study used molecular approaches to determine whether SLNB can be reliably used in patients who have been treated with NAC before SLN surgery, and whether the total tumor load of the SLN can be used as a predictive factor in axillary lymphadenectomy (ALD). We used one-step nucleic acid amplification (OSNA) to analyze a total of 111 consecutive patients who presented operable invasive breast carcinomas and who had been treated with NAC. SLN was positive in 55 patients and the identification rate was 100%. In 9 of these 55 patients, ALD showed that other lymph nodes were also involved. In all of the other 46 patients, the only lymph node to be identified as positive was SLN. Metastasis was not found in any of the axillary lymph nodes in the isolated tumor cell group. The total tumor load, defined as the amount of cytokeratin 19 mRNA copy numbers in all positives SLN (copies/µL), showed three risk groups related to the possibility of positive non-sentinel nodes. OSNA is a diagnostic technique that is highly sensitive, specific, and reproducible and it can be used to analyze sentinel lymph nodes after NAC. Total tumor load may be able to help predict additional metastases in axillary lymphadenectomy.

## 1. Introduction

Sentinel lymph node biopsy (SLNB) is a standard surgical procedure that can be used to diagnose axillary lymph node status in patients with clinically node-negative breast cancer. It is known to be safe and reliable, and has now replaced axillary lymphadenectomy (ALD) [1,2]. It has been pointed out that there is no need for the ALD in patients with low burdens of axillary disease [2,3,4], especially in those who only have micrometastatic disease [4], or if there are only one or two lymph nodes with macrometastases [4].

Neoadjuvant chemotherapy (NAC) is the standard treatment in patients with locally advanced or inflammatory breast cancer, and there is evidence to support its usefulness in the initial stages of the disease [5,6,7,8]. NAC is indicated in patients with tumors of 30 mm or even smaller, when conservative surgery is not possible. NAC has the following advantages: it turns an initial non-surgical breast cancer into one that is operable, it increases the number of conservative surgeries, it evaluates in vivo tumor sensitivity to chemotherapy treatment, it initiates early systemic treatment and it may be useful for translational research. Its potential downsides are that it is a systemic treatment, there is a risk of progression during treatment and the axillary staging is imprecise [5,6,7,8].

This study uses molecular methods to assess whether SLNB can be reliably used in patients treated with NAC before they undergo SLN mapping and surgery. Our findings for SLNB were also correlated with those for axillary lymphadenectomy. The total tumor load (TTL) of the sentinel lymph node results was also evaluated as a predictive factor of positive ALD status in NAC settings.

## 2. Materials and Methods

This prospective and descriptive cohort study was performed in patients with invasive breast carcinomas who had undergone neoadjuvant chemotherapy. The study protocol was reviewed and approved by the Clinical Ethics Committee (CEIC) of the Sant Joan University Hospital in Reus (registration number CEIC14/02/27/2PROJ1), and written informed consent was obtained from each subject in accordance with the 1964 Helsinki declaration and its subsequent amendments.

Between December 2010 and December 2017, one hundred and eleven patients who had been diagnosed with operable invasive breast carcinomas and who had been given neoadjuvant chemotherapy at the Sant Joan University Hospital in Reus underwent breast surgery and an intraoperative axillary lymph node study. The average age of the 111 patients was 55 years, and ranged from 29 to 84 years old. The general criteria for selecting patients were: invasive breast cancer clinical stage T2 and T3, clinically negative preoperative axilla by palpation and axillary ultrasound, cytokeratin 19 expression in breast tumor biopsy, and adequate understanding of surgery and adherence to follow-up standards. The exclusion criterion was negative cytokeratin 19 tumors in the preoperative breast biopsy. Those tumor cells that did not express CK 19 or whose CK 19 expression varied were regarded as being CK 19 negative. To detect SLN metastasis, the SLNs were assayed by one-step nucleic acid amplification (OSNA). The clinicopathological data collected included age, clinical tumor size, pathological tumor size, histological type, nuclear grade, tumor response to chemotherapy (Miller and Payne grading system [9]), axillary lymphadenectomy, estrogen receptor, progesterone receptor, HER2 status and Ki-67 nuclear expression (ICC-4 system for classifying invasive carcinoma). The cases were staged according to the TNM AJCC 8th edition [10].

### 2.1. Sentinel Lymph Node Sampling

A tracer technique was used to carry out the sentinel lymph node procedure for a single tumor and, before surgery, four 0.1 mL deposits were used for peritumoral intra-mammary injection, with a 370 MBq technetium 99 m-labeled sulfur colloid. For multiple tumors, a tracer technique was used to carry out the sentinel lymph node procedure and, before surgery, four 0.1 mL deposits were used for intradermal periareolar injection, with a 370 MBq technetium 99 m-labeled sulfur colloid. Lymphatic mapping was performed on the day before surgery and also just a few hours before. The sentinel node was localized with an intraoperative γ probe. After excision, all active lymph nodes were sent to pathology to be further assessed.

### 2.2. One-Step Nucleic Acid Amplification Assay

Fresh sentinel lymph node specimens were sent to the pathology department. Once the fatty tissue had been cut away, they were weighed and SLN was completely processed by one-step nucleic acid amplification assay. Afterwards, they were homogenized with lysis buffer solution (4 mL) (Lynorhag) and centrifuged at 12,200× *g* at room temperature. An automated gene amplification detection system that uses a reverse transcription loop-mediated isothermal amplification method with RTLAMP was employed to analyze a 2-μL sample of the supernatant and a byproduct of the reaction was used to detect the degree of amplification. After magnesium pyrophosphate had been precipitated, the change in turbidity was correlated with the CK19 mRNA copy number/μL of the original lysate using a standard curve with three calibrators with different CK19 mRNA concentrations. The lymph nodes that weighed more than the specified maximum (600 μg) were cut into two pieces or more and processed separately. As many as four samples were analyzed in one run. Using the calculated number of CK19 mRNA copies per μL, the result was evaluated in terms of the cutoff level calculated by Tsujimoto et al. [11]. The OSNA copy numbers were turned into standard histological measures for lymph node metastasis in the following way: <2.5 × 10^2^ copies/μL of CK19 mRNA were considered to be non-metastasis, between 2.5 × 10^2^ and 5 × 10^3^ copies/μL were considered to be micrometastases, and >5 × 10^3^ copies/μL were considered to be macrometastases. The OSNA assay is sometimes inhibited by inhibitory materials, which results in false-negative (<2.5 × 10^2^ copies/μL) reactions that may be turned into positive (>250 copies/μL) reactions by simple dilution (1:10). However, the values of these reactions after dilution are less reliable for quantitative assessment and were evaluated as + inhibition (+I) (Isolated Tumor Cells (ITC)). The total tumor load (TTL) was calculated in each SLN, and TTL was defined as the amount of CK19 mRNA copies number in all positives SLN (copies/μL). In patients with positive SLN, axillary lymphadenectomy was performed during the same surgical procedure, according to the protocol established in our hospital, which includes axillary lymph node levels one and two.

### 2.3. Statistical Analysis

Baseline characteristics were given as absolute numbers (of patients) and percentages. Ages were compared using the Mann–Whitney test and all other variables with the Pearson Chi-square test. The Z-score was used to search for potential outliers. Confidence intervals (CI) were set at 95%, and *p* < 0.05 was considered to be statistically significant. Logistic regression analysis was used to determine how well the total tumor load at the SLN could explain positive results when non-SLNs were being assessed. Other explanatory variables were also used: age, clinical tumor size, pathological tumor size (pT) post-neoadjuvant treatment, lymphatic invasion (Ly), estrogen receptor (ER) status, progesterone receptor (PgR) status, HER2 status, the number of positive SLNs, the Miller and Payne response index, axillary lymphadenectomy node status by histological examination, and the logarithm of total tumor load mRNA CK19 copy number at the SLNB measured by OSNA (Max CK19 copies). Receiver operating characteristic (ROC) curve analysis and area under the curve (AUC) were used to quantify the prediction performance of each variable. All of the analyses were performed with SPSS version 23 and R version 4.0.

## 3. Results

### 3.1. Patient Characteristics and Pathological Findings

In 104 patients (93.7%), the tumor was invasive carcinoma of no special type and in 7 patients (6.3%) it was special type invasive carcinoma (lobular (2.7%); medullary (1.8%); metaplastic (0.9%); papillary (0.9%). The ICC-4 classification system for invasive carcinoma showed that luminal B was the most common type in 70 patients (63.1%). The next most common type was triple negative in 20 patients (18%), and then HER2 in 16 patients (14.4%) and luminal A in 5 patients (4.5%). The administration of neoadjuvant chemotherapy was carried out following the protocols of our hospital and at the discretion of the medical oncologist. The sequential scheme of chemotherapy consisted of a combination of anthracyclines and taxanes. The anthracyclines used were adriamycin and cyclophosphamide, at doses of 60 mg/m^2^ and 600 mg/m^2^, respectively, and in a regimen of four cycles every 21 days. Paclitaxel and docetaxel were the most widely used taxanes, at doses of 100 mg/m^2^ and 80 mg/m^2^, respectively. Patients over-expressing HER2 tumors received anthracyclines/taxanes and trastuzumab (Herceptin^®^). Trastuzumab was administered in fourteen cycles at 6 mg/m^2^ every 21 days and/or pertuzumab at a total dose of 840 mg, divided into four cycles every 21 days. In addition to the anthracycline/taxane regimen, patients with triple-negative tumors received carboplatin. As far as response to chemotherapy was concerned, pathological response was complete in 38 tumors (34.2%), partial in 67 tumors (60.4%), and non-existent in only 6 tumors (5.4%) (three luminal B type and three triple negative type). The ICC-4 type invasive carcinoma with the best complete pathological response was HER-2 with 13 tumors (81.25%). Follow-up time was 4.81 ± 2.13 years. Overall survival was 93.7% (95%CI 87.0%–97.2%). Breast cancer overall mortality was 3.6% (95%CI 1.2%–9.5%). Disease-free interval did not show a significant difference between the negative and positive sentinel node groups (*p* > 0.005). The comparison between the groups without axillary lymphadectomy and axillary lymphadenectomy did not show significant differences in the disease-free interval (*p* > 0.005). One patient showed axillary recurrence and seven patients died of breast cancer (6.3%) (Table 1).

From the 111 patients, 216 sentinel lymph nodes were obtained and studied by the OSNA assay, an average of 1.96 ± 0.923 SLN/patient. Sentinel lymph nodes of the internal mammary chain were not detected. In 56 patients (50.45%) the OSNA assay was negative. Of the remaining 55 patients (49.55%) with positive SLN, there were 9 (16.36%) ITC (total tumor load (TTL) less than 250 mRNA CK19 copies/μL), 30 (54.55%) micrometastases and 16 (29.09%) macrometastases (TTL between 270 and 2,190,000 mRNA CK19 copies/μL). In 46 of these 55 patients (83.63%), SLN was the only positive lymph node. In two (6.67%) of the patients with micrometastases and in seven (43.75%) of the patients with macrometastases, SLN and axillary lymphadenectomy were positive. No patients with ITC showed axillary lymph node metastases (Table 2).

Each explanatory variable was subject to univariate and multivariate logistic regression analyses to determine the prediction performance of non-SLN metastasis. The results are summarized in Table 3. All parameters are given for both pre- and post-treatment. There was a significant association between the TTL of the sentinel lymph nodes and non-SLN metastasis. The variables in univariate logistic analysis were analyzed with the Cragg–Uhler and McFadden analysis to determine whether they predicted non-SLN status. To prevent multicollinearity, we recognized positive axillary lymph nodes as the only variable related to SLN status (Table 3).

### 3.2. ROC Analysis

**Figure 1:**
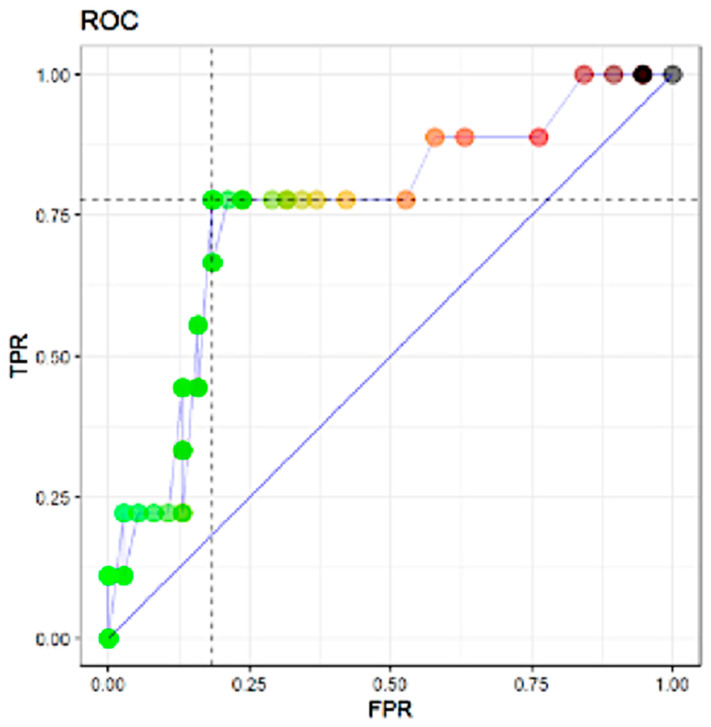
Area under the curve (AUC) 0.7602339. Abbreviations: TPR: true positive rate; FPR: false positive rate

In Figure 1, the results of the ROC analysis for the prediction of non-SLN metastasis include the variables that were shown to be significant (*p* < 0.05) by the univariate logistic Cragg–Uhler and Mc Fadden analyses. In ROC analysis, the TTL of sentinel lymph nodes proved to be the most powerful predictor of non-SLN metastasis by the area under the ROC curve (AUC) (AUC: 0.74 for non-SLN metastasis).

### 3.3. Correlation between Total Tumor Load and Positive Lymph Nodes in Axillar Lymphadenectomy

The risk cohort indicates that the probability of finding positive lymph nodes in axillar lymphadenectomy was significantly increased. The analysis showed that most of the SLNs had similar TTL values (around 20,000 mRNA CK19 copies/μL) and that two patients had relatively low values of 500 mRNA CK19 copies/μL. The group analysis showed three groups in terms of TTL and the probability of lymph node metastasis in axillary lymphadenectomy: (a) a very low-risk group of axillary metastasis, with a TTL between 0 and 500 mRNA CK19 copies/μL without axillary positivity; (b) a low-risk group of axillary metastasis, with a TTL between 501 and 10,000 mRNA CK19 copies/μL, a low probability of axillary positivity (11.11%) and a confidence interval of 1.95, 36.07; (c) a high-risk group of lymph node metastasis, with a TTL greater than 10,000 mRNA CK19 copies/μL, a high probability (50%), and a confidence interval of 26.8, 73.2.

## 4. Discussion

The SLNB technique is now the gold standard for the staging of axillary lymph node status in patients with breast cancer and has become the standard of care to reduce such upper limb morbidity as lymphoedema, shoulder stiffness and chronic pain, which are commonly linked to axillary lymphadenectomy [2,11,12]. One controversial issue is whether SLNB can be useful in patients who have previously been given chemotherapy. According to previous research, primary chemotherapy may modify the patterns of lymphatic drainage in the axilla [13,14,15,16] and the shrinkage of tumors may distort lymphatics if these patterns are aberrant [14,15,16]. Both these cases may affect whether SLNB is detected or not. Studies by individual institutions have reported sensitivity rates of 72–100% and false negative rates of 0–33% when SLNB is performed after neoadjuvant chemotherapy [15,16,17,18,19,20,21,22,23]. In the NSABP B-27 study and the French GANEA study, rates of identification were 85% and 90%, respectively [15,16,17,18,19,20,21,22,23]. In our patients, the rate of identification was similar to that found by previous reports, which suggests that the ability to detect SLN is similar in all patients who have not undergone neoadjuvant chemotherapy.

With regard to the issue of whether analyzing SLNB after chemotherapy might make two surgical procedures unnecessary, the capacity of preoperative chemotherapy to provide a complete pathological response may exploit the down-staging effect of preoperative chemotherapy and reduce the number of patients who need axillary lymphadenectomy. Finally, it does not mean that preoperative chemotherapy can be delayed [17]. In the present study, OSNA was used to analyze all SLNs because it has an advantage over other conventional methods: it can quantitatively assess the TTL in SLNs when the lymph nodes detected are studied. In our study, patients with positive SLNs (ITC, and micro- and macrometastases) underwent axillary lymphadenectomy. Our results revealed that none of the nine patients with ITC had a positive lymph node after axillary lymphadenectomy, that the two patients with micrometastases showed positive lymph nodes after axillary lymphadenectomy, and that seven patients with macrometastases showed additional positive lymph nodes in axillary lymphadenectomy. This finding is interesting because it seems to suggest that, in patients with low TTL, axillary lymphadenectomy might be unnecessary. Our results support the stratification of the TTL and presence of positive lymph nodes in axillary lymphadenectomy into three risk groups: (1) a very low-risk group with a TTL less than or equal to 500 mRNA CK19 copies/μL; (2) a low-risk group with a TTL between 500 and 10,000 mRNA CK19 copies/μL; and (3) a high-risk group with a TTL greater than 10,000 mRNA CK19 copies/μL. Research has shown that the total tumoral load can be used to study SLNs and avoid unwanted surgical procedures [24,25,26,27,28,29,30,31].

The status of the axillary lymph nodes and the internal mammary chain is essential for regional staging and treatment choice. At present, internal mammary chain sentinel lymph node detection remains subject to debate due to ambiguous clinical relevance, and its indications have not been standardized in current guidelines [32] Different studies have shown that in breast cancer, approximately 30% of medial tumors and 15% of lateral tumors show primary lymphatic drainage to the internal mammary chain [33]. Another factor that may affect the detection rate of lymph nodes in the internal mammary chain may be related to the technique of administration of the tracer. Paredes et al. [34], demonstrated an overall detection rate of 14.1%, but the detection rate improved when deep injection, both peritumoral and intratumoral, was used by 17%. Qiu et al. [32] showed that the injection of radiotracer with a modified technique, periareolar intraperenchymal, with high volume and guided by ultrasound, can increase the detection of lymph nodes of the internal mammary chain by 71.1%. In the present study, in patients with breast cancer and neoadjuvant chemotherapy, internal mammary chain drainage was not obtained. The difference in the detection rate between patients without and with NAC could be explained by the effect that NAC produces on the lymphatic drainage pattern [32]. It has been described that NAC could alter the lymphatic drainage patterns due to the contraction to fibrosis of the lymphatic vessels, as well as the obstruction of the lymphatic channels with cellular material or tumor embolisms [32]. However, research in the lymphatic drainages in the mammary chains after NAC have been limited. Studies are required in order to determine the detection capacity of the lymph nodes of the internal mammary chain, in the context of NAC.

Currently, NAC in breast cancer has been accepted as a standard therapeutic procedure for patients with different selection criteria such as: clinical lymph node involvement, tumor size > 2 cm, triple-negative breast cancer, HER2-positive breast cancer, high proliferation index carcinoma, unresectable breast cancer tumors and inflammatory breast carcinoma [35]. Within this group of patients with breast cancer and no axillary lymph node metastases (N0), sentinel lymph node biopsy has been shown to be a safe procedure and can prevent unnecessary axillary lymphadenectomy [36,37,38]. There is no doubt that the efficacy of NAC has shown benefits in terms of overall survival and progression-free survival. Thus, the GANEA 2 study showed that 3-year DFS and OS were 94.8% (95% CI 91%–97.1%) and 97.8% (95% CI 94.9%–99.1%), respectively [36]. Our work showed similar results in terms of overall survival (93.7% (95%CI 87.0%–97.2%)) and breast cancer overall mortality (3.6% (95%CI 1.2%–9.5%)), with a follow-up time of 4.81 ± 2.13 years.

Our study has several limitations. First, the number of patients analysed was not sufficient for firm conclusions to be drawn. Nevertheless, we think that it is still meaningful because we were able to show the accuracy and sensitivity of OSNA in SLN analysis, in the context of NAC. Furthermore, the TTL, assessed by OSNA, can help to predict the likelihood of more axillary metastases in NAC breast cancer. TTL is automatized, reproducible, assessed intraoperatively and not correlated with the type of surgery, and/or the histological tumor subtype and/or neoadjuvant chemotherapy. The high inclusion rate of luminal B invasive carcinoma could be considered a bias, although the patients included represent a real sample of the population in NAC breast cancer. Another clear limitation is that OSNA must be used and it is often not available. However, the use of molecular methods is recommended for better prognosis [10].

## 5. Conclusions

We have demonstrated that whole SLN analysis by OSNA is a highly sensitive, specific and reproducible diagnostic technique in sentinel lymph nodes after clinical node-negative breast cancer patients have undergone neoadjuvant chemotherapy and that TTL can help to predict additional non-SLN metastases in three different risk groups. However, further studies on a larger number of patients are needed to establish a new nomogram, which includes the results of the OSNA assay.

## Figures and Tables

**Table 1:** Characteristics of patients.

Characteristics	No.
Age (years) **	57 (29.5–84.5)
Histological type	
No special type	104 (93.7%)
Special type	7 (6.3%)
Nuclear Grade	
2	44 (39.6%)
3	67 (60.4%)
ICC-4 type	
Luminal A	5 (4.5%)
Luminal B	70 (63.1%)
HER2 positive	16 (14.4%)
Triple negative	20 (18%)
Pathological T classification (pT)	
pT2	91 (81.98%)
pT3	20 (18.02%)
Estrogen receptors **	70 (0–97)
Progesterone receptors **	6 (0–80)
HER-2 expression	
Negative	65 (58.6%)
Positive	46 (41.4%)
Ki-67 **	43 (26.5–64.5)
Pathological response to chemotherapy	
No response	6 (5.4%)
Partial	67 (60.4%)
Complete	38 (34.2%)

Data are summarized as *n* (%), or median [IQR] **, as appropriate.

**Table 2:** Immunocytochemical type (ICC-4), sentinel lymph node and axillary lymphadenectomy (*n* = 111).

	Luminal A	Luminal B	HER2 Expression	Triple Negative
*n*	5	70	16	20
SLN				
Positive	2	41	7	5
Negative	3	29	9	15
SLN positive				
ITC	0	7	1	1
Micrometastases	2	19	6	3
Macrometastases	0	15	0	1
ALD				
Positive	0	8	0	1
Negative	2	33	7	4
Pathological response				
No response	0	3	0	3
Partial response	4	52	3	8
Complete response	1	15	13	9

Abbreviations: SLN: sentinel lymph node; ALD: axillary lymphadenectomy.

**Table 3:** Univariate analysis.

	ALD Negative			ALD Positive			
	Mean	CI lower	CI upper	Mean	CI lower	CI upper	*p* value
Diagnostic age	48.66	45.39	55.35	44.15	40.57	51.40	0.439
Miller–Payne	4	3	5	3	2	3	0.080
Tumor diameter previous treatment (mm)	33	22.25	39.75	43.5	34.5	59.5	0.181
Tumor diameter after treatment (mm)	5	0	0.15	30	12	34	0.026
Total Tumor Load	680,000	352,500	3,875,000	27,410,000	11,530,000	3,000,000	0.043

Abbreviations: ALD: axillar lymphadenectomy.

## Data Availability

Data is contained within the article.
